# Predicting Global Minimum in Complex Beryllium Borate System for Deep-ultraviolet Functional Optical Applications

**DOI:** 10.1038/srep34839

**Published:** 2016-10-13

**Authors:** Qiang Bian, Zhihua Yang, Ying Wang, Chao Cao, Shilie Pan

**Affiliations:** 1 Key Laboratory of Functional Materials and Devices for Special Environments, Xinjiang Technical Institute of Physical & Chemistry, Chinese Academy of Science; Xinjiang Key Laboratory of Electronic Information Materials and Devices, 40-1 South Beijing Road, Urumqi 830011, China; 2 University of Chinese Academy of Sciences, Beijing 100049, China; 3 Department of Physics, Hangzhou Normal University, Hangzhou 310036, China

## Abstract

Searching for high performance materials for optical communication and laser industry in deep-ultraviolet (DUV) region has been the subject of considerable interest. Such materials by design from scratching on multi-component complex crystal systems are challenging. Here, we predict, through density function calculations and unbiased structure searching techniques, the formation of quaternary NaBeBO_3_ compounds at ambient pressure. Among the four low-energy phases, the *P*6_3_/*m* structure exhibits a DUV cutoff edge of 20 nm shorter than α-BaB_2_O_4_ (189 nm) – the best-known DUV birefringent material. While the *P*-6 structure exhibits one time second-harmonic generation efficiency of KH_2_PO_4_ and possesses excellent crystal growth habit without showing any layer habit as observed in the only available DUV nonlinear optical material KBe_2_BO_3_F_2_, whose layer habit limits its wide industrial applications. These NaBeBO_3_ structures are promising candidates for the next generation of DUV optical materials, and the structure prediction technique will shed light on future optical materials design.

Viable DUV (<200 nm) optical materials play a critical role in optical communication and the laser industry^1^,^2^,^3^,^4^,^5^, as they possess wide transparency range and high damage threshold^6^,^7^,^8^,^9^,^10^. Among them, birefringent or nonlinear optical (NLO) materials are utilized in polarization beam splitters, optical isolators, semiconductor photolithography and laser micromachining^11^,^12^,^13^,^14^,^15^. Only a few materials, such as α-BaB_2_O_4_ (α-BBO)^1^,^7^,^8^ and KBe_2_BO_3_F_2_ (KBBF)^16^,^17^, can be practically used as birefringent or NLO materials in the DUV region. However, both α-BBO and KBBF do not have DUV commercial availability since α-BBO exhibits high UV cutoff edge (189 nm) and KBBF suffers from a strong layering tendency. As such, it is the subject of considerable interest to investigate DUV birefringent and NLO materials^18^,^19^,^20^,^21^,^22^. In the past few years, first principle calculations have been an efficient method to evaluate and verify the physical and chemical properties of optical materials^23^,^24^,^25^.

Theoretical search methods can be classified into three categories. The first method used to search optical crystal structure is atom/group/mixing-substitution^26^,^27^,^28^. It comes from the synthetic train of thought of the experimental scientists, and most structure searches of optical materials belong to this method. This method involves a mixing substitution wherein a hypothetical ABX_n_ compound has A-site and B-site substitution in combination with BX_n_ group rotation. The second method is high-throughput computational materials design^29^ by analyzing and calculating large quantities of known candidate structures to discover useful material systems. This method requires a good descriptor that can rapidly characterize relative properties, through using this descriptor to search new materials in enormous data repositories. This method has been successfully used for mid-infrared NLO materials^30^ and scintillators^31^,^32^.

Although the above two methods have offered some useful suggestions to experimental synthesis, they do not provide the lowest energy structures on the free energy surface for an undiscovered crystal structure, which are closely related to experimental results. Is there an efficient means to address this issue? The third method provides the suitable solution-crystal structure prediction, which aims at finding the global minimum on a potential energy surface. Structure prediction has been widely applied in high pressure regimes^33^,^34^, material surface reconstruction^35^ and cluster structure search^36^. However, owing to the complexities of optical material systems, which are always more than binary system, no functional optical material structure prediction has been reported.

Here we first introduce a systematic global structure optimization method to develop the next generation of DUV optical materials in complex beryllium borate quaternary system that is attributable to its very short absorption edges. Through searching free energy surfaces (more than 40,000 structures) via the newly developed Artificial Bee Colony (ABC) algorithm as implemented in the CALYPSO (crystal structure analysis by particle swarm optimization) code^37^,^38^, we have extensively explored the stable phases of NaBeBO_3_ at ambient pressure, and obtained a comprehensive set of structures for NaBeBO_3_. In particular, we have established for the first time the thermodynamically stable structures. In doing so, the first example of 3-fold coordinated Be atoms in borates is observed among the energetically favorable NaBeBO_3_ structures. Equally important, the four lowest energy NaBeBO_3_ structures possess superior linear and nonlinear optical properties, as well as good chemical stability. They are attractive candidates for the next generation of DUV birefringent or DUV NLO materials. Our studies also provide insights and a road map for exploring the design and synthetic strategies for NaBeBO_3_. Such research also offers a key route to structural determination of materials with specific functional optical properties.

## Results

### Crystal structures

**Figure 1:**
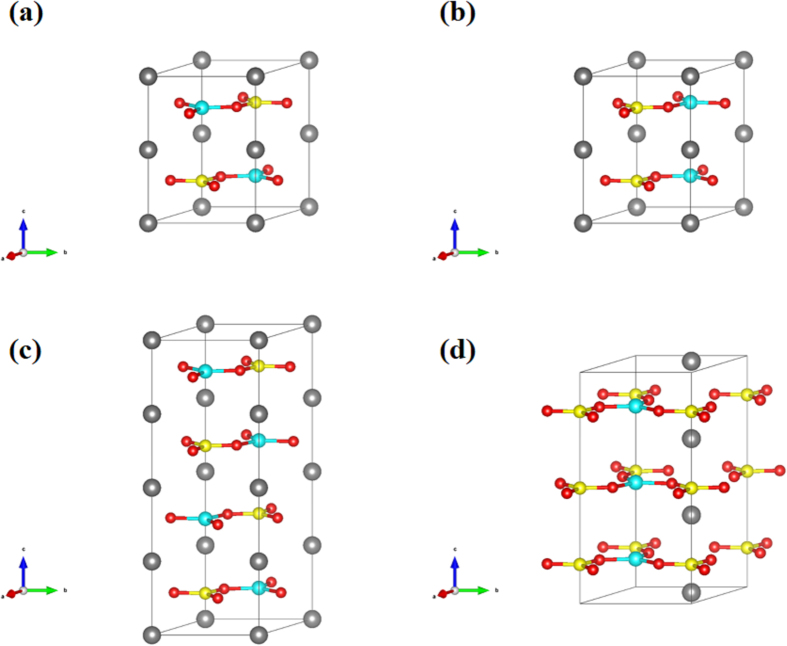
Predicted structures of NaBeBO_3_ at ambient pressure. The gray, blue, yellow and red balls represent Na, Be, B and O atoms, respectively. (**a**) *P*6_3_/*m* structure. (**b**) *P-*6*c*2 structure. (**c**) *P-*3*c*1 structure. (**d**) *P-*6 structure. Structural details are given in the Supporting Information.

Structure predictions through the ABC technique in the CALYPSO code with simulation sizes up to six NaBeBO_3_ formula units (f.u.) per simulation cell were performed twice at ambient pressure. The four structures with lowest energy were systematically studied, and the other five structures with higher energy are shown in Supplementary Fig. 1 and Supplementary Table 1. The four energetically favorable structures belong to hexagonal *P*6_3_/*m* (2f.u. per cell, Fig. 1a; Supplementary Table 1), hexagonal *P-*6*c*2 (2f.u. per cell, Fig. 1b; Supplementary Table 1), trigonal *P-*3*c1* (4f.u. per cell, Fig. 1c; Supplementary Table 1) and hexagonal *P-*6 (3f.u. per cell, Fig. 1d; Supplementary Table 1) space group. Two of the energetically favored structures are non-centrosymmetric, i.e. hexagonal *P-*6*c*2 and *P-*6. In the four NaBeBO_3_ structures, *P*6_3_/*m*, *P-*6*c*2, *P-*3*c1* and *P-*6, the planar BeO_3_ and the BO_3_ units connect with each other through corner-sharing the O atoms to generate a (BeBO_3_)_∞_ layer. This layer is perfectly parallel to the *ab* plane. One (BeBO_3_)_∞_ layer links with an adjacent layer through six-coordinate Na atoms. As shown in Fig. 1(a–d), the difference of the NaBeBO_3_ structures is the direction and position of the planar BeO_3_ units and BO_3_ units in the (BeBO_3_)_∞_ layer. In addition, the Na-O, Be-O and B-O distances are in the range of 2.432–2.452, 1.543–1.547 and 1.384–1.386 Å, respectively. The O-B-O and O-Be-O angles are all 120° to make the planar BO_3_ and BeO_3_ units to be equilateral triangles. Intriguingly, to the best of our knowledge, it is the first time to discover the 3-fold coordinated Be atoms in borates, similar to the Be atoms in Y_2_BeO_4_ (ref. 39) and Rb_2_Be_2_Si_2_O_7_^40^.

### Structural stability

**Figure 2:**
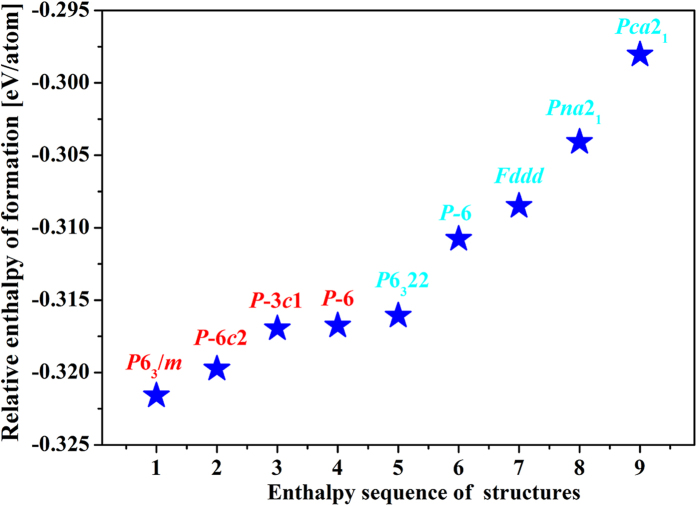
Relative enthalpies of formation per atom with respect to Na_2_O, B_2_O_3_ and BeO for different NaBeBO_3_ structures at ambient pressure.

Formation enthalpies can be used to assess relative stability of crystalline compounds at low temperatures. The formation enthalpy for NaBeBO_3_ can be defined as follows:


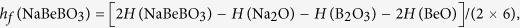


where *h*_*f*_ is the enthalpy of formation per atom and *H* is the calculated enthalpy per chemical unit for each compound at ambient pressure, here, Na_2_O, B_2_O_3_ and BeO are chosen as the reference structures attributed to their stability at ambient pressure. The formation enthalpies of the NaBeBO_3_ phases, calculated at higher levels of accuracy, are plotted in Fig. 2. The phase with space group of *P*6_3_/*m* is the most stable structure with the formation enthalpy about −0.322 eV/atom, the remaining phases with space groups *P-*6*c*2, *P-*3*c1* and *P-*6 have formation enthalpies of about 2 meV/atom, 5 meV/atom and 5 meV/atom higher than that of the *P*6_3_/*m* structure. The formation enthalpies (Supplementary Table 2) of several other reaction paths are also negative, these results indicate that the NaBeBO_3_ structures might be experimentally synthesizable. The phonon calculations have also shown that these structures are kinetically stable by reason that none of the imaginary phonon mode exists in the whole Brillouin Zone (Supplementary Fig. 2).

### Chemical bondings

**Figure 3:**
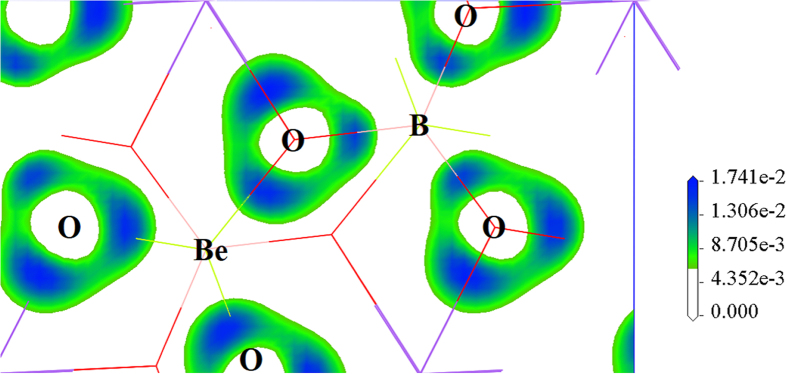
Isosurface of ELF for (BeBO_3_)_∞_ layer in *P*6_3_/*m* structure.

Owing to the unexpected discovery of the triangular BeO_3_ units in NaBeBO_3_, a detailed bonding analysis was performed. The nature of this bonding was probed by calculating the electron localization functions (ELF) (Fig. 3; Supplementary Fig. 3), which is known to be an effective tool to distinguish different bonding interactions in solids^41^. In the NaBeBO_3_ structures, the Na-O bonds are ionic, and the B-O bonds are covalent, that is in accordance with other borates. In particular, the Be atoms in the NaBeBO_3_ structures are *sp*^2^ hybridized and form σ bonds with their neighboring O atoms. These Be-O bonds are reasonable that the Be-O distances (1.54–1.55 Å) are in the range of the reported Be-O σ bonds (1.51–1.87 Å) in Y_2_BeO_4_ and Rb_2_Be_2_Si_2_O_7_. These conclusions are further substantiated by Mulliken population analysis^42^ listed in Table 1, where the calculated overlap populations for Na-O, B-O and Be-O are 0.03, 0.87 and 0.64 e, respectively, indicating the ionic character of the Na-O bonds and the covalent nature of the B-O and Be-O bonds.

### Electronic structures

**Figure 4:**
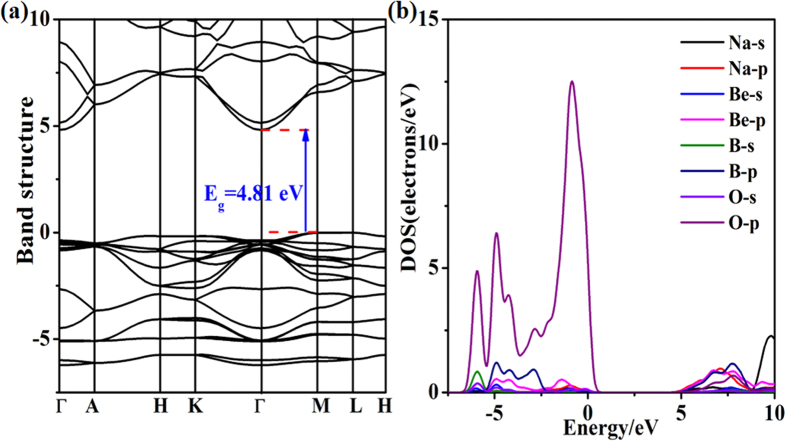
Band structure and density of states of *P*6_3_/*m* structure.

The calculated electronic band structures (Fig. 4; Supplementary Fig. 4) for the NaBeBO_3_ structures reveal that the phases with space groups *P*6_3_/*m*, *P-*6*c*2, *P-*3*c1* and *P-*6 are all indirect band gaps, which are 4.81, 4.80, 4.75 and 4.73 eV by the PBE functional and 7.32, 7.30, 7.25 and 7.24 eV by the PBE0 hybrid functional (Supplementary Fig. 5), respectively. The latter functional has been shown to be more precise for band gap calculations in borates^43^,^44^. Our calculated band gaps by the PBE0 hybrid functional are in good agreement with experimental and earlier theoretical predictions of sodium beryllium borates^45^.

Since the electronic transitions from occupied states to unoccupied states reveal the microcosmic origin of the optical properties, it is essential to analyze the partial density of states (PDOS) of the NaBeBO_3_ structures in detail. The NaBeBO_3_ structures have much similar electronic states distribution near the band gap. The top region of the valence band (VB) is mainly occupied by the 2s orbitals of the boron atoms and the 2p orbitals of the boron, beryllium and oxygen atoms. The B 2s, 2p states and the Be 2p states show a wide hybridization with O 2p states from −7 to 0 eV, indicating that the B-O and Be-O bonds, that belong to the (BeBO_3_)_∞_ layers, make the main contribution to the top of the VB in the NaBeBO_3_ structures. The bottom region of the conduction band (CB) is mainly occupied by the 2p states of Na, Be, B and O atoms. It is clear that the B-O and Be-O groups in the (BeBO_3_)_∞_ layers are the main contributor to the optical properties. In addition, because of the small contribution of Na to the top of the VB and the bottom of the CB, its impact on the optical properties is negligible.

### Birefringent crystal material candidates

**Figure 5:**
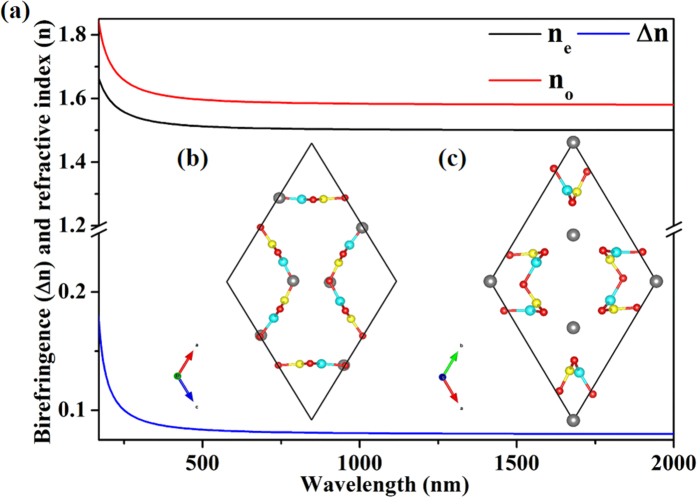
(**a**) Refractive indices and birefringence of *P*6_3_/*m* structure. (**b**) The first simulative *P*6_3_/*m* NaBeBO_3_ structure. (**c**) The second simulative *P*6_3_/*m* NaBeBO_3_ structure. The gray, blue, yellow and red balls represent Na, Be, B and O atoms, respectively.

The calculated refractive indices and birefringence of the NaBeBO_3_ structures are shown in Fig. 5a and Supplementary Fig. 6. Because their point groups belong to hexagonal or trigonal crystal system, they are all uniaxial crystals with the birefringence values equal to |*n*_*e*_−*n*_*o*_|. The birefringence values of the phases with space groups *P*6_3_/*m*, *P-*6*c*2, *P-*3*c1* and *P-*6 are 0.0825, 0.083, 0.085 and 0.084 at 589.3 nm, respectively. Their large birefringence, which is related to their strong optical anisotropy, derives from the parallel arrangement of the (BeBO_3_)_∞_ layers. The calculated charge densities (Supplementary Fig. 7) show large electron density anisotropy for the (BeBO_3_)_∞_ layers, supporting our argument that the (BeBO_3_)_∞_ layers are the major contributors to the optical anisotropy.

Interestingly, the NaBeBO_3_ structures are the first kind of negative uniaxial borate crystal structure type to produce large birefringence summarized by us^46^, that is effectively fundamental building units in a parallel arrangement with a high number density. To further verify this conclusion, two simulated NaBeBO_3_ structures with space group *P*6_3_/*m* (Fig. 5b,c; Supplementary Table 3), that belong to the second kind of positive uniaxial borate crystal structure type to induce large birefringence, are chosen to investigate the optical anisotropy. In the two simulated NaBeBO_3_ structures, the planar BO_3_ and BeO_3_ groups form equilateral triangle arrangements and are parallel to the b or c axis. The birefringence values (Δn = n_e_ − n_o_; Supplementary Fig. 8) of the simulated structures are 0.0850 and 0.0813 at 589.3 nm, respectively. The large birefringence values of the phases with space group *P*6_3_/*m* and two other simulated *P*6_3_/*m* structures well validate the two large birefringence structure type in uniaxial borates.

The UV cutoff edges of the phases with space groups *P*6_3_/*m*, *P-*6*c*2, *P-*3*c1* and *P-*6 are 169.4 (7.32 eV), 169.9 (7.30 eV), 171.0 (7.25 eV) and 171.2 nm (7.24 eV) respectively, which are 20, 20, 18 and 18 nm shorter than that of α-BBO (189 nm). That is to say, these compounds have large birefringence and short deep UV cutoff edges, which demonstrate that they are excellent candidates for birefringent materials.

### Nonlinear optical material candidates

**Figure 6:**
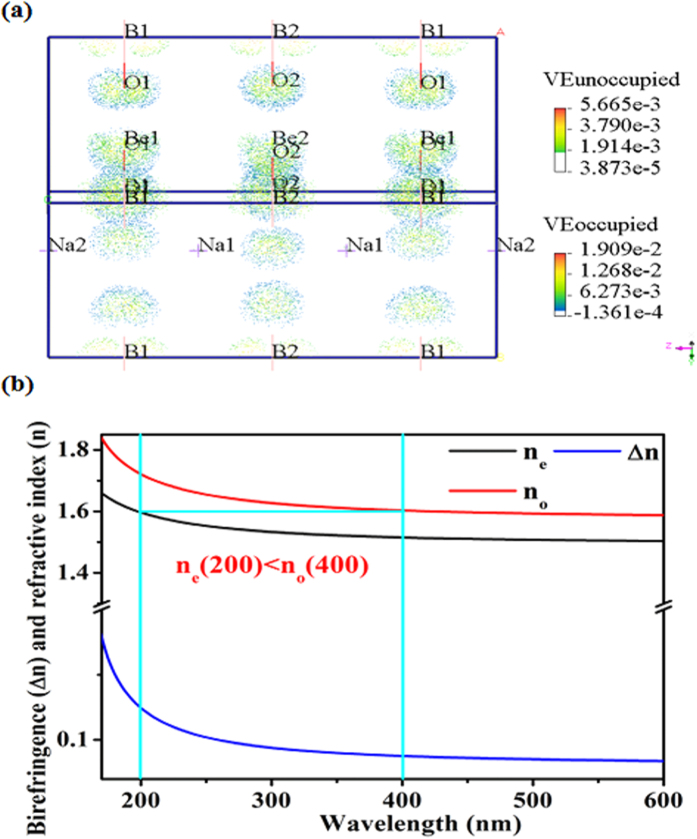
(**a**) Occupied states and unoccupied states in the d_22_ SHG-densities of the *P-*6 structure in the VE process. (**b**) Refractive indices and birefringence of *P-*6 structure, and the phase-matching capabilities for *P-*6 at 400 nm. In negative uniaxial crystal, the n_e_ at 200 nm located between the n_e_ and n_o_ refractive index at 400 nm indicates the achievement of the phase-matching condition.

The second-harmonic generation (SHG) effects are estimated owing to the absence of inversion symmetry in the phases with space groups *P-*6*c*2 and the *P-*6. The calculated SHG coefficients are *d*_21_ = −*d*_22_ = *d*_16_ = −0.07 pm/V for the *P-*6*c*2 structure, which is only 1/5 times that of KH_2_PO_4_ (KDP, *d*_36_ = 0.39 pm/V). For the *P-*6 structure, the coefficients are *d*_11_ = −*d*_12_ = −*d*_26_ = 0.07 pm/V and *d*_22_ = −*d*_21_ = *d*_16_ = −0.40 pm/V, the d_22_ coefficient contributes to the effective SHG coefficients (Φ = 90° for type-I phase matching), that is equivalent to that of KDP. The SHG-weighted electron densities (Fig. 6a; Supplementary Fig. 9), where only the virtual-electron attributed to its large contribution (more than 90%), indicate that the BeO_3_ and BO_3_ groups are mainly responsible for second-order NLO effects.

A marked difference in the SHG response is found in the *P-*6*c*2 and the *P-*6 structures. Their structures are similar that the BO_3_ and the BeO_3_ units in adjacent (BeBO_3_)_∞_ layers are almost in opposite directions, that is considered to weaken the SHG effects. Then what is the key to the notable SHG difference? The answer is the number of (BeBO_3_)_∞_ layers in the primitive cell, the *P-*6*c*2 phase has two (BeBO_3_)_∞_ layers, whereas the *P-*6 phase has three (BeBO_3_)_∞_ layers. As no additional layer counteracts the third layer’s SHG effect, the *P-*6 phase exhibits superior NLO properties to the *P-*6*c*2 phase.

In negative uniaxial crystal, the SHG phase-matching condition can be summarized as follows: n_e_(*λ*/2) ≤ n_o_(*λ*), where n_e_(*λ*/2) and n_o_(*λ*) are the corresponding refractive index at the SHG and fundamental wavelengths, respectively. The calculated refractive index (Fig. 6b; Supplementary Fig. 10) reveals that in the *P-*6*c*2 and the *P-*6 phases n_e_(200 nm) < n_o_(400 nm), indicating that these two NaBeBO_3_ structures achieve the SHG phase-matching condition in the DUV region. The shortest SHG wavelengths for the *P-*6*c*2 and *P-*6 are all about 195 nm.

**Figure 7:**
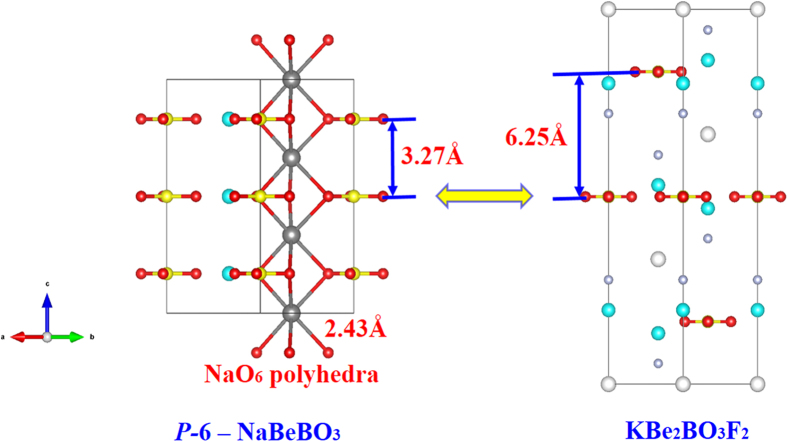
The interlayer spacing of the *P*-6 NaBeBO_3_ (left) and KBe_2_BO_3_F_2_ structure (right). The NaO_6_ polyhedra between the (BeBO_3_)_∞_ layers in *P*-6 NaBeBO_3_ structure are plotted in the left. The gray, blue, yellow, red, white and light gray balls represent Na, Be, B, O, K and F atoms, respectively.

Equally important, good crystal growth habit is essential to DUV functional optical material. It is well known that KBBF is the only material to generate coherent light in the DUV region by direct SHG response, whereas its layer habit seriously impedes its commercialization. The interlayer spacing of the *P-*6*c*2 and *P-*6 phases (about 3.27 Å; Fig. 7) is 2.98 Å shorter than that of KBBF (6.25 Å). Their adjacent (BeBO_3_)_∞_ layers are connected by the NaO_6_ polyhedra (about 2.43 Å), whose interlayer binding forces far outweigh that of KBBF. This confirms the reduction of layering tendency in *P-*6*c*2 and *P-*6.

## Discussion

By using the newly developed ABC structure searching algorithm and density functional total energy calculations, we systematically studied the possible existing phases of the NaBeBO_3_ quaternary compound at ambient pressure. The *sp*^2^ hybrid 3-fold coordinated Be atoms, which are never found in borates before, are discovered in four lowest energy NaBeBO_3_ structures. More importantly, the four lowest energy NaBeBO_3_ structures have short DUV cutoff edges about 170 nm and reduce the layer habit, and they exhibit extraordinary optical properties. The large birefringence values show that the NaBeBO_3_ structures are excellent candidates for DUV birefringent materials. The electronic structures and the SHG-weighted electron densities clearly show that the parallel (BeBO_3_)_∞_ layers are the main origin of the optical properties in these energetic favorable NaBeBO_3_ structures. On the whole, the *P-*6 phase exhibits a short UV cutoff edge of 171 nm (7.24 eV), a good SHG efficiency about one time that of KH_2_PO_4_, and is capable of being phase-matcheble in the DUV region. Importantly, it mitigates the laying tendency as observed in KBBF. All these facts demonstrate that NaBeBO_3_ with *P-*6 space group is a promising DUV NLO material. In summary, this work represents a significant step forward in predicting global optimization structures of complex crystal system, and also provides a successful manner to search for the next generation of DUV functional optical materials.

## Methods

Before exploring the title compound, several known functional optical materials were predicted in the same manner. The experimental structures of KBe_2_BO_3_F_2_^47^, BPO_4_^48^, AgGaS_2_^49^, ZnGeP_2_ (ref. 50) were all successfully reproduced at ambient pressure through ABC technique, validating our methodology in application to functional optical materials. The results are listed in Supplementary Fig. 11 and Supplementary Table 4.

### Crystal structure prediction

We obtain candidate structures of NaBeBO_3_ by using swarm-intelligence CALYPSO (Crystal structure AnaLYsis by Particle Swarm Optimization) structure searching simulations unbiased by any prior known structure knowledge and in conjunction with first-principles density functional calculations. The artificial bee colony (ABC) algorithm, which is originally attributed to Karaboga *et al.*^51^, with symmetry constraint is implemented into CALYPSO software to keep the structural symmetry during structure evolution. The remarkable feature of this methodology is the capability of predicting stable structure with the only knowledge of the chemical composition.

We searched for structures of NaBeBO_3_ with simulation cell sizes of 1–6 formula units (f.u.) at ambient pressures using the CALYPSO package^52^. The local structural relaxations calculations were performed in the framework of density functional theory, using the generalized gradient approximation^53^ within the Perdew-Burke-Ernzerhof exchange functional theory^54^, and the frozen-core projector-augmented wave (PAW) method as implemented in the VASP code^55^. The adopted PAW pseudopotentials of Na, Be, B and O treat *3s*^1^, *2s*^2^, *2s*^*2*^*2p*^*1*^ and *2s*^*2*^*2p*^*4*^ electrons as valence. The cutoff energy 900 eV and appropriate Mnokhorst-Pack k-meshes were chosen to ensure that all enthalpy calculations were well converged to better than 1 meV/atom. The phonon calculations have been carried out by using a supercell approach as implemented in the PHONOPY code^56^. More computational details can be found in the Supplementary Information.

### Properties calculation

After ranking the enthalpy order of the NaBeBO_3_ structures, the electronic band structure, DOS and optical property calculations of the four low enthalpy structures were performed within the framework of DFT within the generalized gradient approximation (GGA) in the scheme of Perdew–Burke–Ernzerhof^54^, as implemented by CASTEP code^57^. The norm-conserving pseudopotentials used treat *2s*^*2*^*2p*^*6*^*3s*^*1*^, *2s*^*2*^, *2s*^*2*^*2p*^*1*^ and *2s*^*2*^*2p*^*4*^ as valence electrons for Na, Be, B and O atoms, respectively. The cutoff energy 810 eV and Monkhorst-Pack *k* point meshes with a grid of 0.025 Å^−1^ for Brillouin zone sampling were chosen to achieve a well-converged electronic structure and optical properties.

PBE0 hybrid functional^58^ was used to calculate the band structures. The calculation of optical properties was scissor corrected by the calculated energy gap difference between the PBE and the PBE0, the scissor operators of the four lowest energy structures are 2.51 eV (*P*6_3_/*m*), 2.50 eV (*P-*6*c*2), 2.50 eV (*P-*3*c1*) and 2.50 eV (*P-*6). The SHG-weighted electron densities analysis^59^ was used to analyze NLO response of the NaBeBO_3_ structures. The theoretical methods were applied with success in a previous study to analyze the SHG coefficients of the NLO crystals^60^.

## Additional Information

**How to cite this article**: Bian, Q. *et al.* Predicting Global Minimum in Complex Beryllium Borate System for Deep-ultraviolet Functional Optical Applications. *Sci. Rep.* **6**, 34839; doi: 10.1038/srep34839 (2016).

## Supplementary Material

Supplementary Information

## Figures and Tables

**Table 1:** Bond distances and Mulliken overlap populations for the characteristic atomic pairs in four lowest energy NaBeBO_3_ structures.

Value atomic pair	Bond distances (Å)	Overlap populations (e)
Na-O	2.432–2.455	−0.03
B-O	1.384–1.386	0.87
Be-O	1.544–1.547	0.64
