# Supplementary material for: Predicting Global Minimum in Complex Beryllium Borate System for Deep-ultraviolet Functional Optical Applications
Source: Sci Rep. 2016 Oct 13;6:34839. doi: 10.1038/srep34839 (PMC5062158; doi:10.1038/srep34839)
Supplement: Supplementary Information [file srep34839-s1.pdf]

Supplementary Information for:

Predicting Global Minimum in Complex Beryllium Borate System for Deep-ultraviolet Functional Optical Applications

Qiang Bian[1,2], Zhihua Yang[1*], Ying Wang[1], Chao Cao[3], Shilie Pan[1*],

[1]*Key Laboratory of Functional Materials and Devices for Special Environments Xinjiang Technical Institute of Physical & Chemistry, Chinese Academy of Science; Xinjiang Key Laboratory of Electronic Information Materials and Devices, 40-1 South Beijing Road, Urumqi 830011, China;*

[2]*University of Chinese Academy of Sciences, Beijing 100049, China*

[3]*Department of Physics, Hangzhou Normal University, Hangzhou 310036, China.*

Supplementary Figures

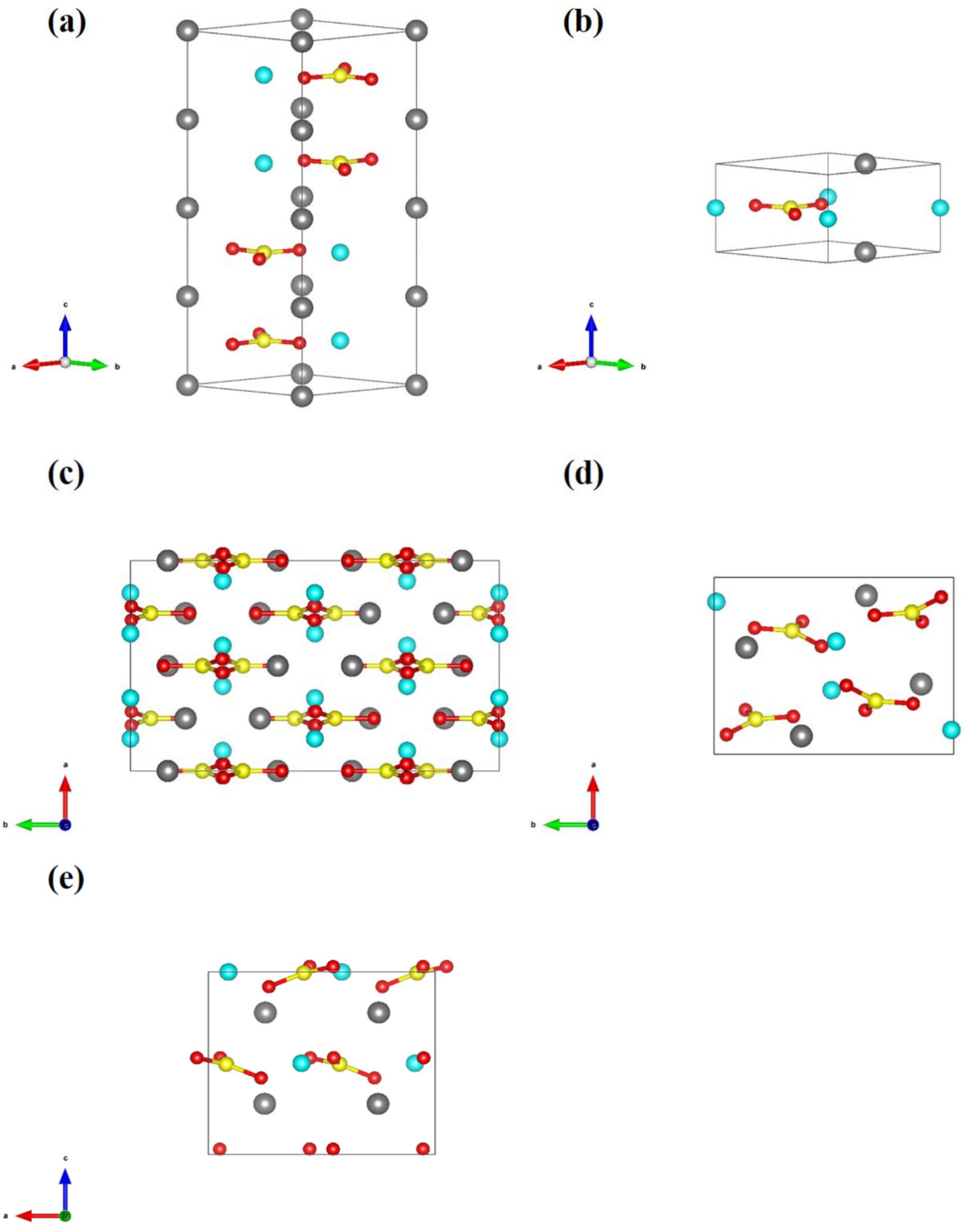

Supplementary Figure 1. Predicted structures of $NaBeBO_3$ with higher energy at ambient pressure. The gray, blue, yellow and red balls represent Na, Be, B and O atoms, respectively. (a) $P6_322$ structure. (b) P-6 structure. (c) *Fddd* structure. (d) $Pna2_1$ structure. (e) $Pca2_1$ structure.

In order to check the dynamic stability of the nine lowest energy $NaBeBO_3$ structures, the phonon spectra were calculated at ambient pressure. No imaginary modes for these structures are found at ambient pressure.

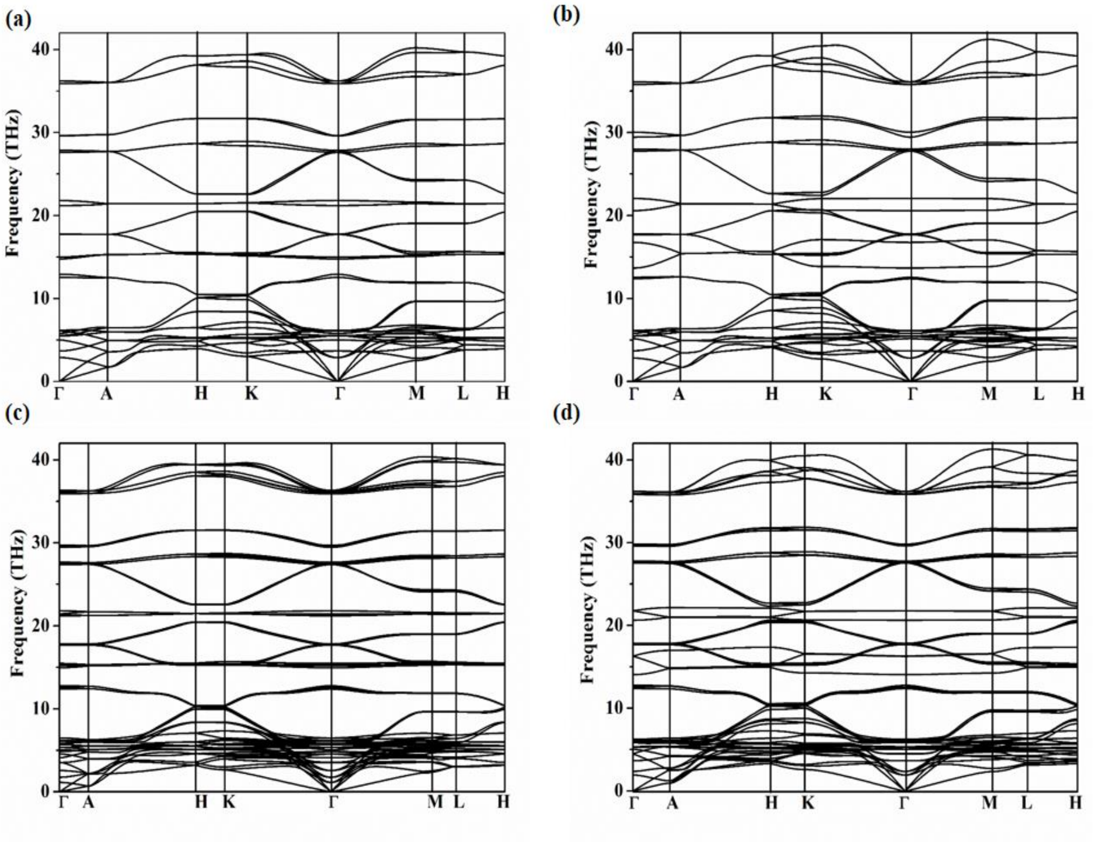

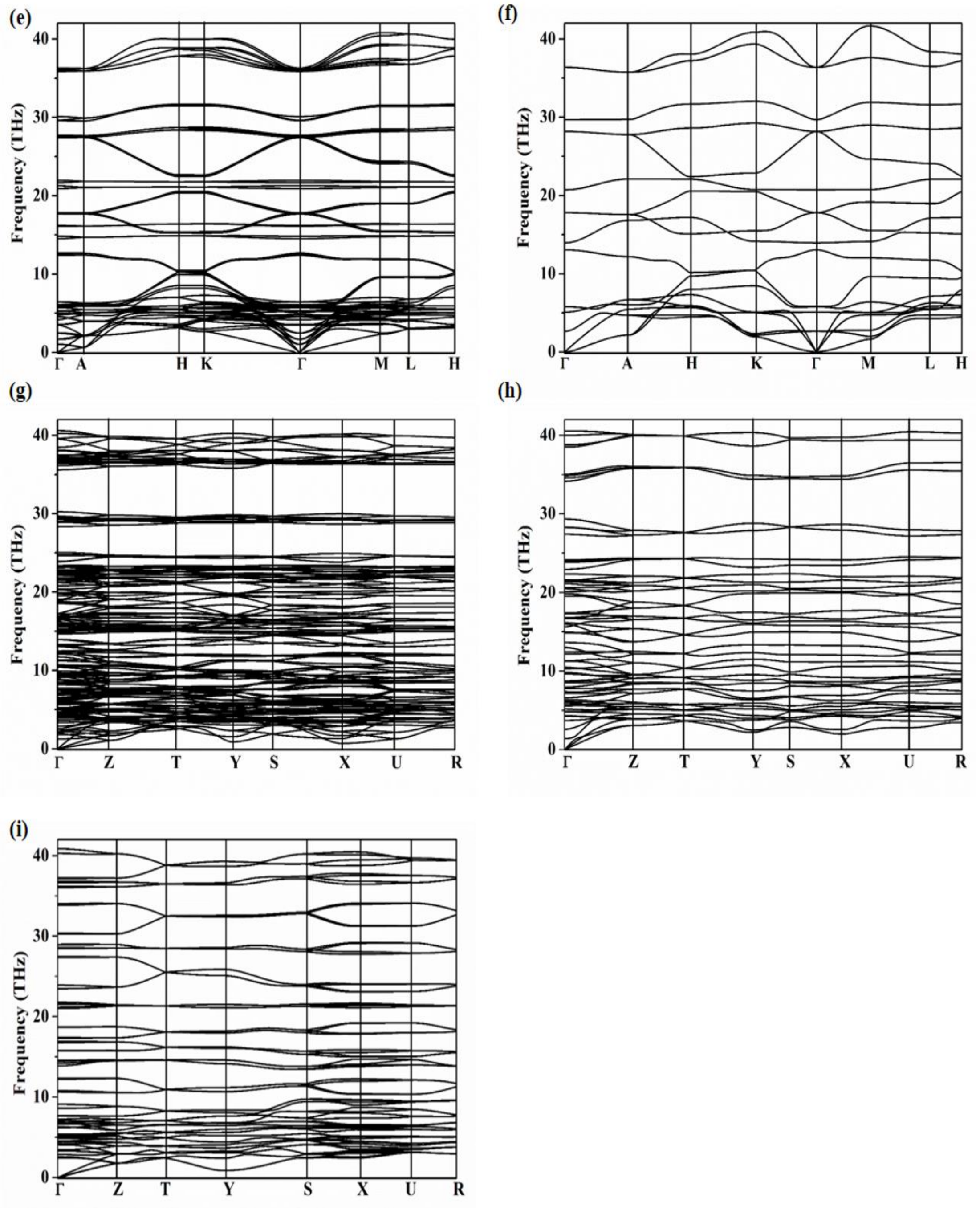

Supplementary Figure 2. Calculated phonon band structures of the $NaBeBO_3$ structures: $P6_3/m$ (a), P-6c2 (b), P-3$c1$ (c), P-6 (d), $P6_322$ (e), P-6 (f), $Fddd$ (g), $Pna2_1$ (h) and $Pca2_1$ (i) (same order with Supplementary Table 1).

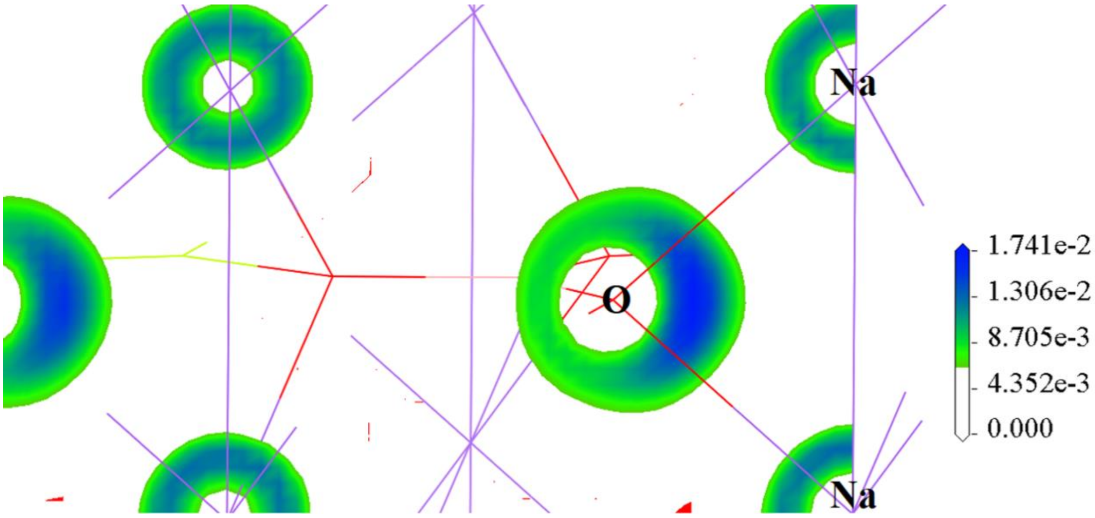

Supplementary Figure 3. Isosurface of ELF for Na-O bonds in $P6_3/m$ structure.

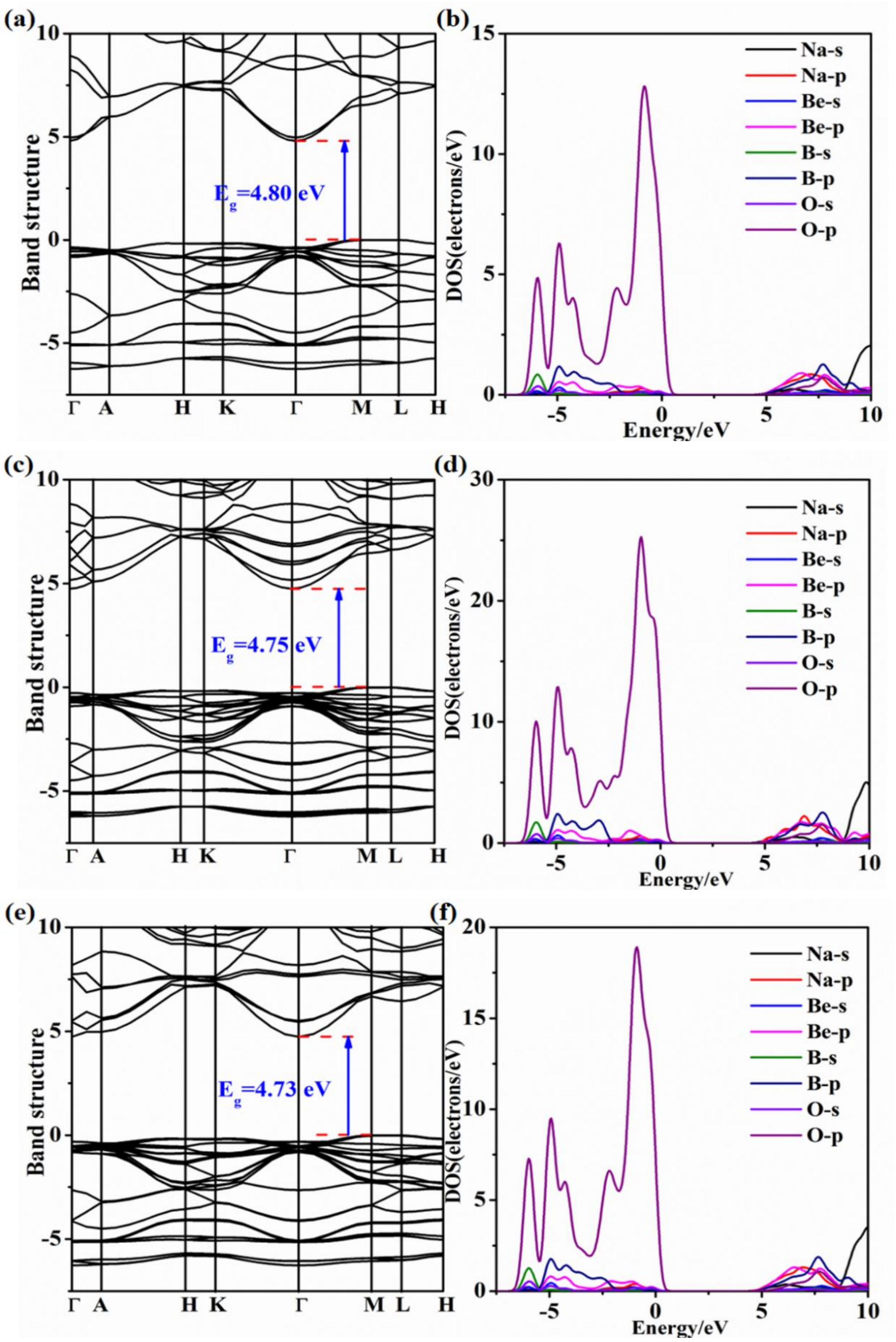

Supplementary Figure 4. Band structure and density of states of *P*-6*c*2 (a and b), *P*-3*c*1 (c and d), *P*-6 (e and f) structures by the PBE functional.

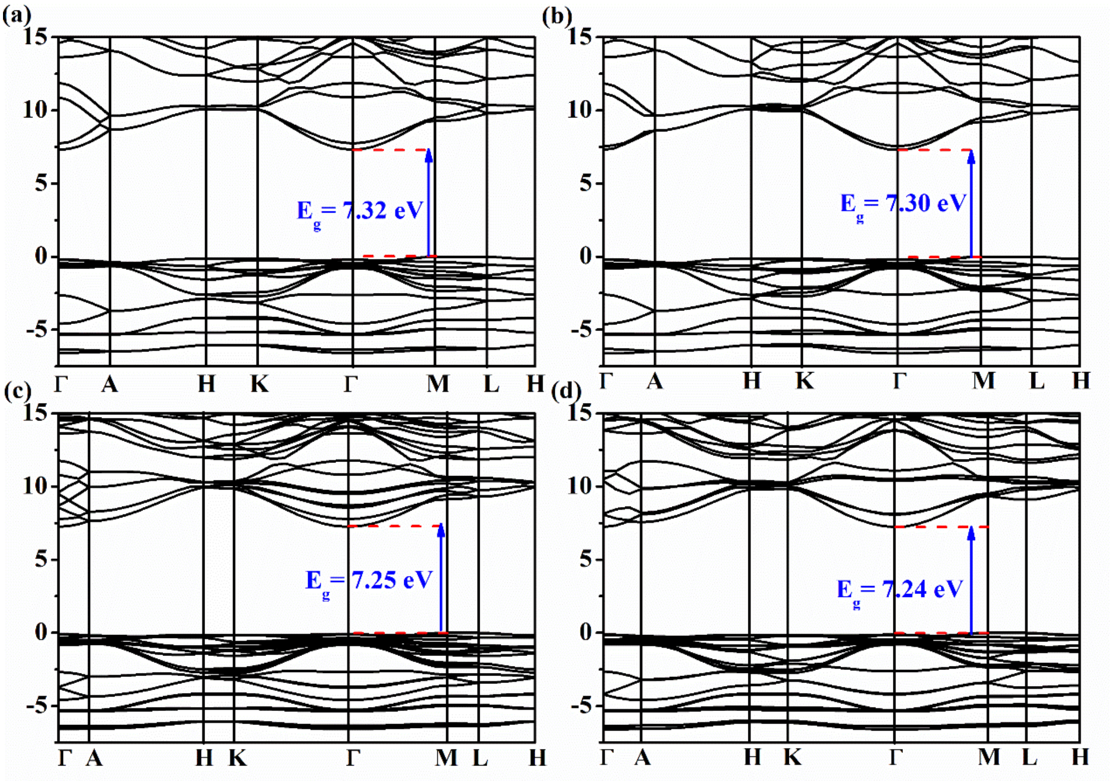

Supplementary Figure 5. Band structure of $P6_3/m$ (a), *P*-6*c*2 (b), *P*-3*c*1 (c) and *P*-6 (d) structures by the PBE0 functional.

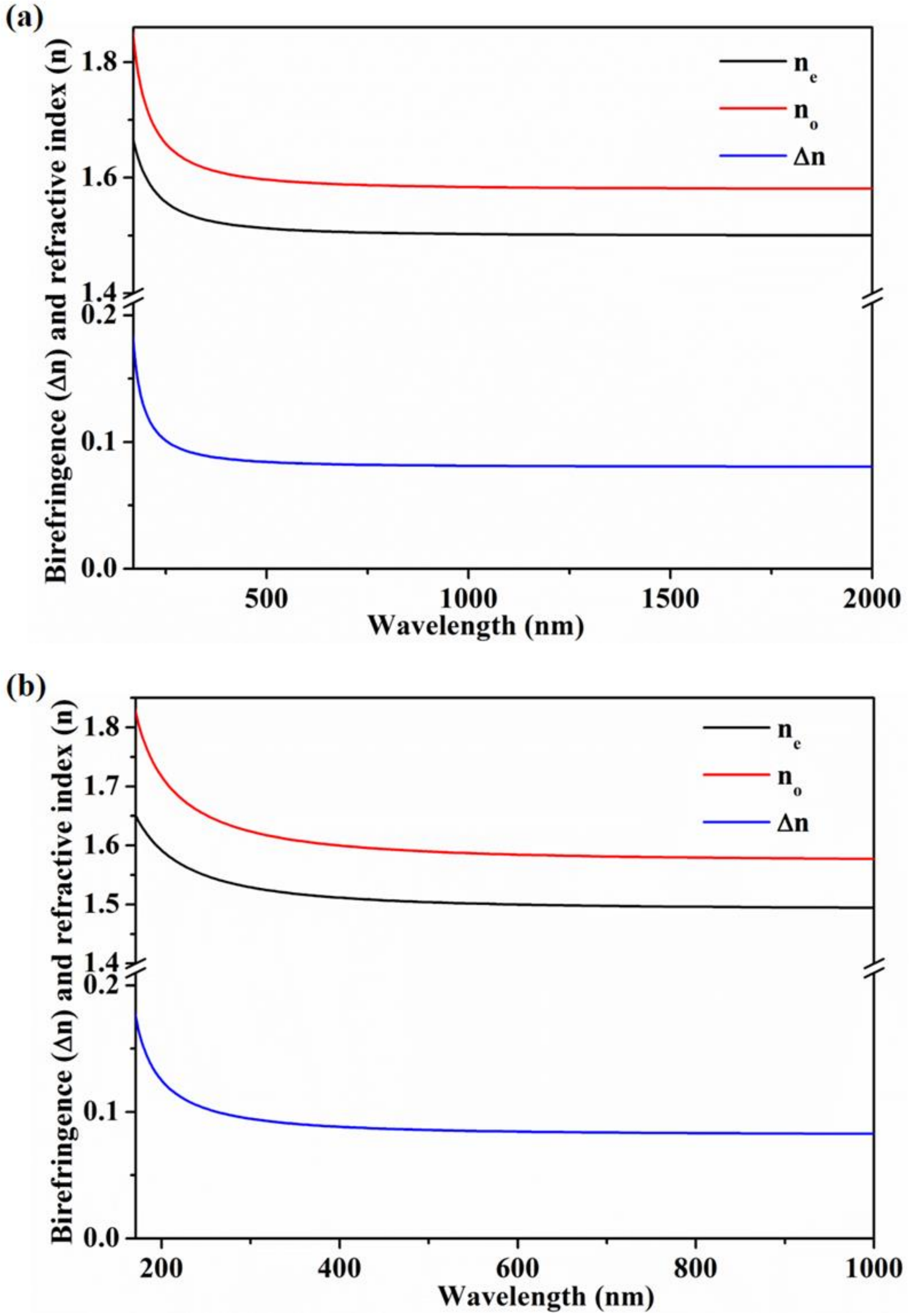
(a)
n_e
n_o
Δn
1.8
1.6
1.4
0.2
0.1
0.0
Birefringence (Δn) and refractive index (n)
500
1000
1500
2000
Wavelength (nm)
(b)
n_e
n_o
Δn
1.8
1.7
1.6
1.5
1.4
0.2
0.1
0.0
Birefringence (Δn) and refractive index (n)
200
400
600
800
1000
Wavelength (nm)

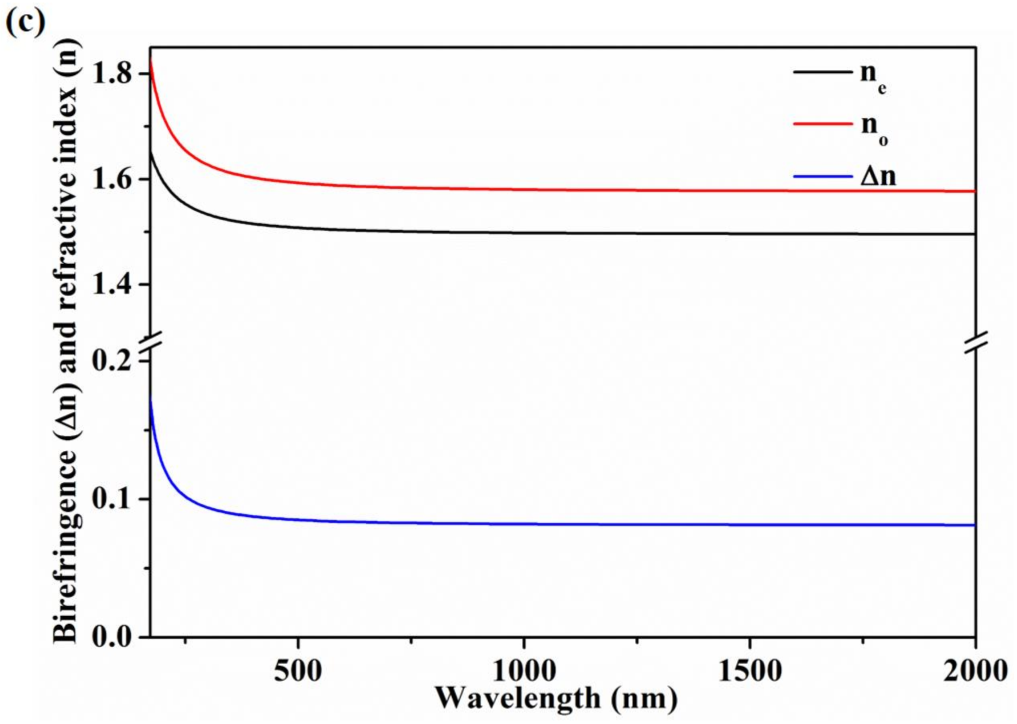

Supplementary Figure 6. Refractive indices and birefringence of *P*-6*c*2 (a), *P*-3*c1* (b) and *P*-6 (c) structures.

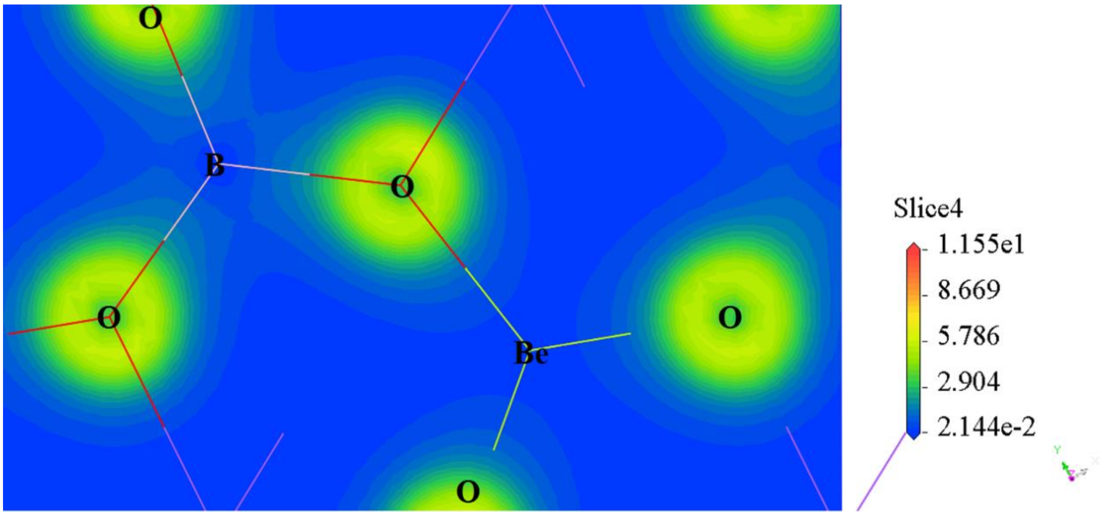

Supplementary Figure 7. Calculated charge density of $(BeBO_3)_\infty$ Layer in $P6_3/m$-$NaBeBO_3$ structure.

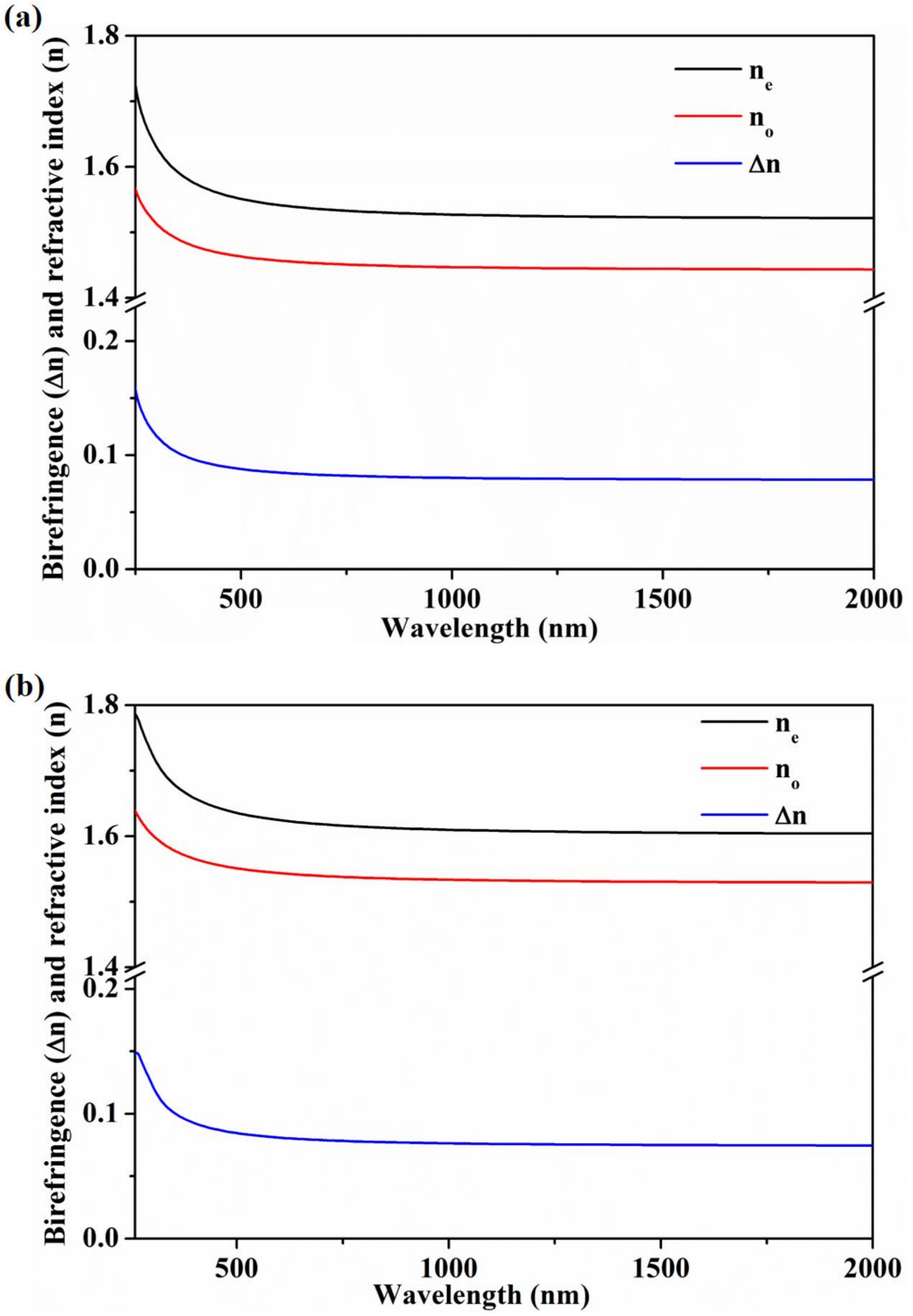

Supplementary Figure 8. Refractive indices and birefringence of two simulated $P6_3/m$-$NaBeBO_3$ structures.

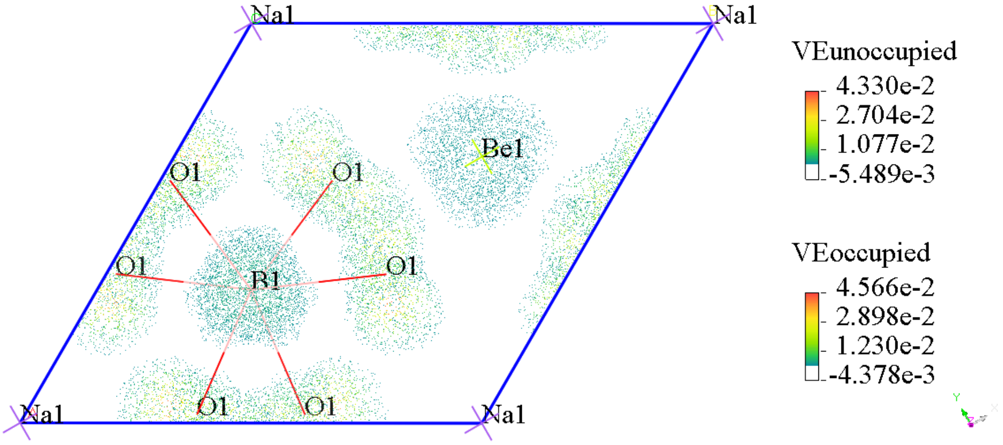

Supplementary Figure 9. Occupied states and unoccupied states in the d_{22} SHG-densities of the *P*-6c2 structure in the VE process.

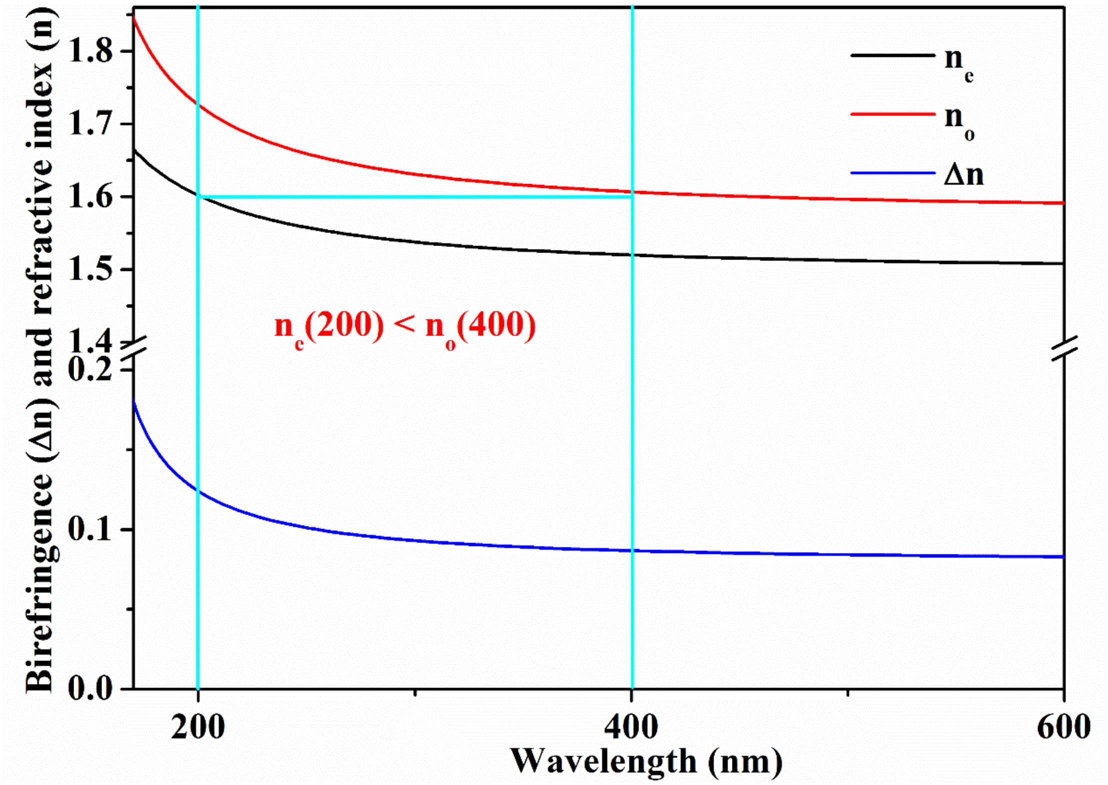

Supplementary Figure 10. Refractive indices and birefringence of *P*-6*c*2 structure, and the phase-matching capabilities for *P*-6*c*2 at 400 nm.

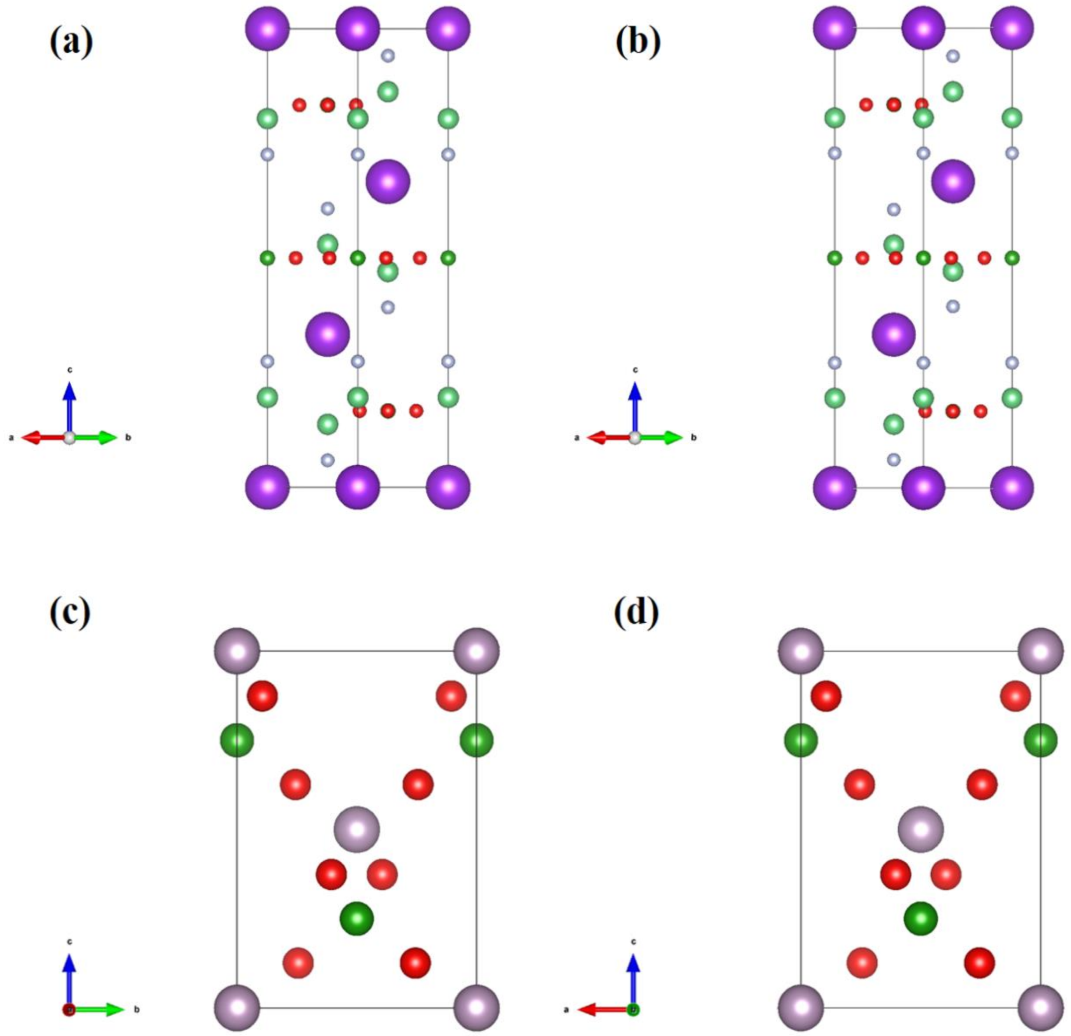
(a)
(b)
(c)
(d)

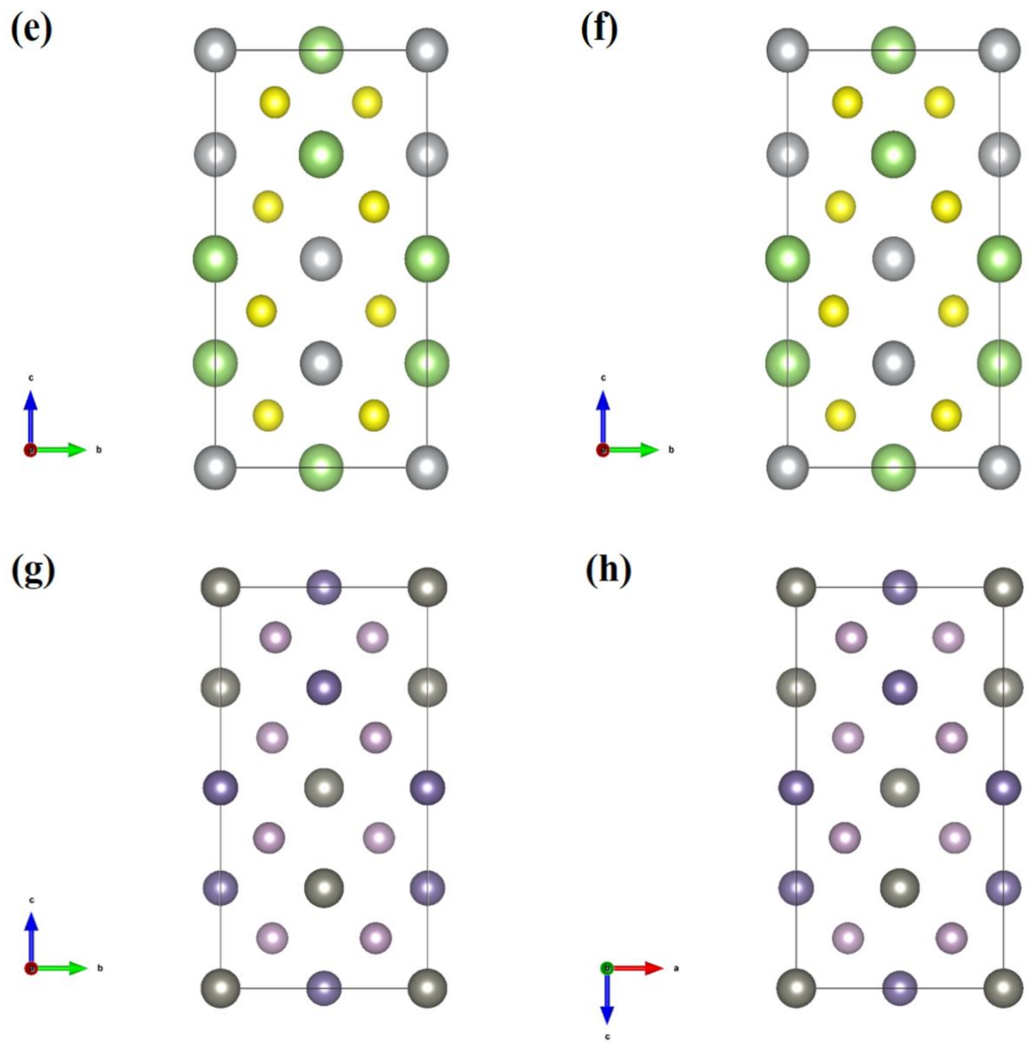

Supplementary Figure 11. (a), (c), (e), (g) represent experimental $KBe_2BO_3F_2$, BPO_4, $AgGaS_2$, $ZnGeP_2$ structures, and (b), (d), (f), (h) represent the predictive structures, respectively.

Supplementary Tables

Supplementary Table 1. Structure parameters and enthalpy values of $NaBeBO_3$ structures.

Space group	Enthalpy values (eV/atom)	Lattice parameters	Atom	Site	Atomic positions
$P6_3/m$	-0.32160	a=b=4.7053	Na	2b	0.00000 0.00000 0.00000
		c=6.5210	Be	2c	0.33333 0.66667 0.25000
			B	2d	0.33333 0.66667 0.75000
			O	6h	0.37239 0.97859 0.75000
P-6*c*2	-0.31974	a=b=4.7021	Na	2a	0.00000 0.00000 0.00000
		c=6.5379	Be	2d	-1.66667 -0.33333 0.25000
			B	2f	-1.33333 -0.66667 0.25000
			O	6k	-0.37246 -0.39355 0.75000
P-3*c1*	-0.31696	a=b=4.7015	Na	2a	0.00000 0.00000 0.00000
		c=13.1477	Na	2b	0.00000 0.00000 0.00000
			Be	4d	0.33333 0.66667 0.12331
			B	4d	0.33333 0.66667 0.87646
			O	12g	0.62774 0.02134 0.12360
P-6	-0.31677	a=b=4.7009	Na	2h	0.33333 0.66667 0.66534
		c=9.8822	Na	1c	0.33333 0.66667 0.00000
			Be	2i	0.66667 0.33333 0.16931
			Be	1f	0.66667 0.33333 0.50000
			B	2g	0.00000 0.00000 0.16921
			B	1b	0.00000 0.00000 0.50000
			O	3k	0.72690 0.68771 0.50000
			O	6l	0.72688 0.03915 0.16922
$P6_322$	-0.31608	a=b=4.7012	Na	2a	1.00000 0.00000 0.00000
		c=13.1932	Na	2b	1.00000 0.00000 0.25000
			Be	4f	0.66667 0.33333 0.62576
			B	4f	0.33333 0.66667 0.62649
			O	12i	0.62761 0.02120 0.37383
P-6	-0.31608	a=b=4.6971	Na	1c	0.33333 0.66667 0.00000
		c=3.34009	Be	3b	0.00000 0.00000 0.50000
			B	1f	0.66667 0.33333 0.50000
			O	3k	0.94013 0.64563 0.50000
Fddd	-0.30850	a=7.56026	Na	16f	0.00000 0.10095 0.00000
		b=13.26537	Be	16e	0.65360 0.00000 0.00000
		c=9.85069	B	16f	0.50000 0.19518 0.00000
			O	32h	0.96731 0.25236 0.88116
			O	16f	0.00000 0.40990 0.00000

$Pna2_1$	-0.30409	a=6.16542	Na	4a	0.89670 0.36442 0.48286
		b=8.36083	Be	4a	0.36235 0.50944 0.47864
		c=4.58648	B	4a	0.80162 0.17477 0.93807
			O	4a	0.24882 0.36904 0.65742
			O	4a	0.71377 0.83064 0.52079
			O	4a	0.88882 0.05211 0.12174
$Pca2_1$	-0.29459	a=7.966	Na	4a	0.24827 0.74118 0.77709
		b=4.7768	Be	4a	0.41115 0.17959 0.99939
		c=6.6696	B	4a	0.07870 0.32258 0.99466
			O	4a	0.23262 0.24070 0.91891
			O	4a	0.44958 0.86317 0.02899
			O	4a	0.05288 0.60621 0.03007

Supplementary Table 2. The formation enthalpies of other reaction pathways for *P*6_3/*m*-$NaBeBO_3$ structure.

Reaction pathway	Formation enthalpy (meV/atom)
$Na_2B_4O_7$ + Na_2O + 4BeO – 4$NaBeBO_3$	-122.945
α-$Na_2O(B_2O_3)_3$ + 2Na_2O + 6BeO – 6$NaBeBO_3$	-211.536
$Na_4B_2O_5$ + B_2O_3 + 4BeO – 4$NaBeBO_3$	-50.301
α-$Na_2B_8O_{13}$ + 3Na_2O + 8BeO – 8$NaBeBO_3$	-204.953
Na_2BeO_2 + B_2O_3 + BeO – 2$NaBeBO_3$	-179.314
$Na_6Be_8O_{11}$ + Na_2O + 4B_2O_3 – 8$NaBeBO_3$	-213.413

Supplementary Table 3. Structure parameters of two simulated $P6_3/m$–$NaBeBO_3$ structures.

Space group	Lattice parameters	Atom	Site	Atomic positions
$P6_3/m$-a	a=b=11.0615	Na	6h	-0.39506 -0.38797 -0.25000
	c=5.0292	Be	6h	-0.26170 0.14427 -0.25000
		B	6h	-0.40518 -0.13325 -0.25000
		O	6h	0.00253 0.60660 -0.25000
		O	12i	-0.40958 -0.19632 -0.48673
$P6_3/m$-b	a=b=9.9227	Na	2b	0.00000 0.00000 0.00000
	c=5.0556	Na	2d	0.33333 0.66667 0.75000
		Na	2c	0.66667 0.33333 0.75000
		Be	6h	0.65496 0.86400 0.75000
		B	6h	0.84313 0.64286 0.75000
		O	6h	0.81103 0.02238 0.75000
		O	12i	0.21047 0.77793 0.48699

Supplementary Table 4. The structure information of experimental and predictive $KBe_2BO_3F_2$, BPO_4, $AgGaS_2$, $ZnGeP_2$ structures in detail.

	Space group	Lattice parameters (Å)	Volume of conventional cell(Å^3)	Difference of predictive and experimental volume
Experimental $KBe_2BO_3F_2$	*R*32	a=b=4.38172 c=19.16167	318.606	2.5%
Predictive $KBe_2BO_3F_2$		a=b=4.38535 c=19.60529	326.521	
Experimental BPO_4	*I*-4	a=b=4.55093 c=6.71412	139.056	0.3%
Predictive BPO_4		a=b=4.55812 c=6.71499	139.515	
Experimental $AgGaS_2$	*I*-42*d*	a=b=5.76107 c=10.71053	355.481	0.09%
Predictive $AgGaS_2$		a=b=5.76433 c=10.70884	355.828	
Experimental $ZnGeP_2$	*I*-42*d*	a=b=5.69743 c=11.10653	360.526	-0.04%
Predictive $ZnGeP_2$		a=b=5.69941 c=11.09399	360.370	

Supplementary Methods

Details of crystal structure predictions. Our artificial bee colony (ABC) structural prediction approach is based on a global minimization of free energy surfaces merging *ab initio* total-energy calculations with CALYPSO methodology as implemented in the CALYPSO code.[1,2] This method is designed to predict the stable structures of given compounds with only the knowledge of chemical compositions at given condition, such as pressure. The structures of stoichiometry $NaBeBO_3$ (NBBO) were searched twice with simulation cell sizes of 1-6 formula units (f.u.) at ambient pressure. The first generation was produced randomly, each generation contained 50-60 structures. Local optimizations used the VASP code[3,4] and stopped when Gibbs free energy changes became smaller than 1×10^{-5} eV per cell. In the next CALYPSO runs, 60% of lowest-enthalpy structures were utilized to produce the structures in the next generation by ABC algorithm, and the rest 40% structures were generated randomly. The structural searching simulation for each run was stopped after 2000-3600 structures (40-60 generations) were obtained.

To further analyze the structures with higher accuracy, a number of low-enthalpy structures were selected and optimized using density functional theory within the generalized gradient approximation[5] as implemented in the VASP code. The plane wave cutoff energy is set to 900 eV in all calculations, and the Monkhorst-Pack *k*-mesh with a maximum spacing of

0.18 Å^{-1} was employed to ensure the enthalpy values are well converged to less than 1 meV/atom. The phonon calculations were performed by using the finite displacement approach[6] as implemented in the Phonopy code.

Supplementary references

1. Wang, Y. C., Lv, J., Zhu, L. & Ma, Y. M. Crystal structure prediction via particle swarm optimization. *Phys. Rev. B* **82,** 094116 (2010).

2. Wang, Y. C., Lv, J., Zhu, L. & Ma, Y. M. CALYPSO: a method for crystal structure prediction. *Comput. Phys. Commun.* **183,** 2063-2070 (2012).

3. Kresse, G. & Hafner, J. *Ab initio* molecular dynamics for liquid metals. *Phys. Rev. B* **47,** 558 (1993).

4. Kresse, G. & Furthmüller, J. Efficient iterative schemes for *ab initio* total-energy calculations using a plane-wave basis set. *Phys. Rev. B* **54,** 11169 (1996).

5. Perdew, John P. *et al.* Atoms, molecules, solids, and surfaces: Applications of the generalized gradient approximation for exchange and correlation. *Phys. Rev. B* **46,** 6671, (1992).

6. Parlinski, K., Li, Z. Q. & Kawazoe, Y. First-principles determination of the soft mode in cubic ZrO_2. *Phys. Rev. Lett.* **78,** 4063 (1997).
